# Doxorubicin Loaded DNA Aptamer Linked Myristilated Chitosan Nanogel for Targeted Drug Delivery to Prostate Cancer

**Published:** 2017

**Authors:** Fereshteh Atabi, Seyed Latif Mousavi Gargari, Mehrdad Hashemi, Parichehreh Yaghmaei

**Affiliations:** a *Department of Biology, Science and research branch, Islamic Azad University, Tehran, Iran. *; b *Department of Biology, Faculty of Science, Shahed University, Tehran, Iran.*; c *Department of Genetics, Tehran Medical Sciences branch, Islamic Azad University, Tehran, Iran.*

**Keywords:** DOX, MCS, Nanogel, Aptamer, Targeted drug delivery, Prostate cancer

## Abstract

Recently, specific attention has been paid to aptamers, short DNA or RNA, as a tool for cancer diagnosis and therapy. In the present study MCS nanogels were prepared by Myristate: Chitosan at 1:9 ratio and were characterized by several techniques. A selected ssDNA aptamer (Apt) capable of detecting LNCaP cells was linked to Myristilated Chitosan nanogels (Apt-MCS) by glutaraldehyde and loaded with Doxorubicin (DOX) to be used in targeted drug delivery against the Prostate cancer cells. LNCaP and PC-3 cells were treated with Apt-MCS-DOX complex and the binding efficiency was estimated by flow cytometry. The binding affinity of the selected aptamers was above 70% compared to the initial library. The loading capacity of the nanogel was as high as 97% and up to 40% of DOX were released from MCS within 15 days. Cytotoxicity of nanodrug on LNCaP cells was determined by MTT assay. Apt-MCS-DOX was specifically binded to LNCaP cells whereas it didn’t show any specificity to PC-3 cells as a negative control. Both MCS-DOX and Apt-MCS-DOX showed a lethal effect on LNCaP cells. Our results can lead to an aptamer based simple and applicable technique for early diagnosis and treatment of cancerous cells.

## Introduction

Prostate Cancer (Pcanc) is the second cause of cancer death in men and the most common malignant cancers after Lung cancer (1). So far, basal treatment of Pcanc relies on surgery, chemotherapies and ionizing radiation, with a low efficacy and high toxicity to healthy tissues (2). Therapeutic systems with their tumor targeting ability that specifically allows delivery of drugs to the cancerous cells, strongly increases the therapeutic consequences while minimize unspecific toxicity (3). Nanomedicine is a strong, long lasting option for prevention of Pcanc associated with decreasing mortality and minimal nonspecific side effects (4). Many kinds of multifunctional nano particles as carriers of drugs, have been provided to promote drug delivery (5). Drug targeting can be obtained by conjugation of certain ligands such as aptamers or antibodies developed against a specific molecule on target cell (6). Aptamers, single-stranded RNAs or DNAs, are chemically synthesized and have high affinity and specificity to a variety of targets such as ATP, proteins, peptides, amino acids, antibiotics, small chemicals, viruses, whole cells, receptors, antigens or even metal ions compared to the antibodies and enzymes (7, 8, 9). Aptamers may act better than antibodies in discovery of new biomarkers, *in-vitro* and *in-vivo* diagnosis, controlled drug release, and targeted therapy (9). Cell-specific aptamers in the detection of cancerous cells with their rapid penetration into tissues without any immunogenicity makes them valuable tool with numerous applications in research and medical therapeutic purposes (10). They can easily distinguish between two different cancerous cells or between cancerous and normal cells (11). Aptamers are synthesized independent of biological systems and are generated in the laboratory by a chemical process named “Systematic Evolution of Ligands by Exponential enrichment” (SELEX) (12). 

Due to its biocompatibility, nonimmunogenicity, high stability and low toxicit, Chitosan (CS) currently has been used for delivery systems (5). Glucosamine units of CS have provided the hydroxy and amino groups as the reactive sites for crosslinking reactions. The cross-linkers can modify the properties of Chitosan hydrogels and cross-linked hydrogels can absorb a huge amounts of different kinds of biological fluids or water (13). CS can be modified to increase its solubility at physiological pH or to facilitate its endosomal escape, by improving its ‘proton-sponge’ capacity (14). In the present study, partially Myristilated CS was used for preparation of MCS nanogel to improve the controlled drug release system.

 The nanogel was loaded with Doxorubicin, the most important cytostatic drugs used in the field of cancer therapy. It has considerable activity against tumor of the ovaries, lung, testes, prostate, cervix, bladder and Ewing’s carcinoma (15). *In-vitro* loading and release of nanohybrid MCS-DOX was investigated on the bases of MCS: DOX ratio. Morphological characterization of the MCS nanogel beads was studied by Scanning Electron Microscopy (SEM), Fourier-transform infrared spectroscopy (FT-IR), and Dynamic Light Scattering (DLS). Simultaneously, we developed an aptamer from a DNA aptamer library against LNCaP cells by a whole cell-SELEX method. In order to arrive at a targeted drug delivery system, the aptamer was conjugated with MCS nanogel. The specificity of the complex of aptamer-MCS nanogel for the LNCaP cells was further investigated using PC-3 cells as a control.

## Experimental

*Materials*

Chitosan, Glycine, Sodium borohydride (NaBH_4_)_,_ Penicillin/ Streptomycin (Pen Strep), Myristic acid, EDC [1-ethyl-3 (3-dimethylaminopropyl)Carbodiimide], NHS (N-hydroxysuccinimide), MTT[*3-(4,5-dimethylthiazol-2-yl) -2,5-diphenyltetrazolium bromide*] and Rhodamin 123 were from Sigma Aldrich (USA). Glacial acetic acid (100%), DMSO (Dimethyl Sulfoxide), BSA (Bovine serum albumin) were from Merck (Germany). Doxorubicin hydrochloride (DOX-HCl) was from EBEWE PharmaGes (Austria). Agarose and Low Melting Agarose were from Banglor Genei India Co. RPMI 1640 medium and fetal bovine serum (FBS) were from Gibco (Invitrogen). The LNCaP and PC-3 cell lines were from the Pasteur Institute of IRAN. All other chemicals used in this research were of analytical grade from Merck (Germany) or Sigma Aldrich (USA).

*Buffers*

DPBS (pH 7.2-7.4): CaCl_2_.2H_2_O 0.1 g, Glucose 0.5 g, KCl 0.2 g, KH_2_PO_4_ 0.1 g, MgSO_4_.7H_2_O 0.1 g, Na_2_HPO_4_ 0.05 g and NaCl 4 g.

Wash buffer (WB): DPBS 500 mL, Glucose 2.25 g and MgCl_2_ 1M 2.5 mL.

Binding Buffer (BB): DPBS 500 mL, Glucose 2.25g, MgCl_2_ 1M 2.5 mL and BSA. Keep in 4 °C for 1 month.

*Cell Culture*

The LNCaP cells as a positive target and PC-3 cells as a negative control were cultured in RPMI 1640 under standard cell culture conditions supplemented with 10% FBS and 1% Pen Strep/ 37 ^o^C in a humid environment with 5% CO_2_. 

*Primers and Library*

The forward primer with the following sequence was labeled at the 5′-end with –NH2 for conjugation of aptamer to nanogel. 

5`-NH2-CATCCATGGGAATTCGTCGA 3`

The reverse primer with the following sequence was labeled at the 5′ end with phosphate for selective digestion of the phosphorylated strand of double stranded DNA (dsDNA) from the 5’ end of PCR products by an exodeoxyribonuclease enzyme (Lambda exonuclease) as described by Marimuthu (13). 

5`-p- CTGCCTAGGCTCGAGCTCG 3`

The initial library with the following sequence were designed and obtained from the GSFLX Company (German). 

5` CATCCATGGGAATTCGTCGAC (N)_ 40_ CGAGCTCGAGCCTAGGCAG 3`

*Cell-SELEX Procedure*

Method of Kang (16) with some modifications was used for Cell-SELEX. In brief, in each round of selection, 5 nmol of ssDNA aptamers per 1mL BB, was first folded by incubation/ 95^ o^C for 10 min and immediately cooled on ice for 15 min. ssDNA aptamers was incubated with approximately 10^6 ^live washed LNCaP cells with gentle shaking/ room temperature (RT). After 1 h, the cells containing the binding aptamers were collected with centrifugation/ 1000 RPM for 3 min/ 4^ o^C and washed with WB. The pellet was heated/ 95^ o^C for 5 min in 500 µL DNAase-free water and the supernatant containing aptamers were collected and amplified by PCR. The PCR product was treated with Lambda exonuclease and the concentration of ssDNA was determined by a nanodrop. Five nmol of the ssDNA was used for the second round of SELEX. The steps were repeated for 10 rounds of SELEX with gradually increasing the stringency of the reaction by decreasing the incubation time from 1 h to 15 min and increasing washing buffer volume from 1 mL to 10 mL. 

 In order to eliminate the nonspecific binding aptamers to common antigens, the counter SELEX was carried out with the incubation of the DNA aptamers obtained from the 3^rd^ and 7^th^ rounds of SELEX with 10^6 ^live PC-3 cells. The cells were precipitated and the supernatant containing the unbounded aptamers was amplified and used in further SELEXes. 

*Cloning and Sequencing of Enriched Pools*

Enriched aptamers with high affinity to LNCaP cells were collected, PCR amplified and were cloned into the pTG19-T plasmid and was transformed into *E*.*coli*. Positive clones resulting from transformation were selected and the affinity of the candidate aptamers to LNCaP cells was assayed separately. Two of the clones with highest affinity were sequenced. 

The binding affinity of the finally selected aptamer was estimated by flow cytometry. Target cells (1 × 10^6^) were added to varying concentrations of FITC-labeled aptamers (0, 50, 100, 150, 200 pmol) in a 200 μL volume of BB. The original library was used as a negative control. The dissociation constant (K_d_) was estimated by the equation Y = B max X/ (K_d_ + X), using Sigma Plot 12.0 software (Jandel, San Rafael, CA).

*Preparation of Myristylated Chitosan (MCS) Nanoparticles*

A homogeneous solution was prepared by dissolving 0.250 g Chitosan powder in 50 mL acetic acid aqueous solution (2%) and stirred for 20 min/room temperature (RT). The 0.5% (w/v) solution of CS was sonicated with the power of 70W for 20 min/18 ^o^C. CS nanogels was cross linked with Myristate using the EDC / NHS technique described by Cheng *et al. *(18). CS nanogels (5 mg/mL) was mixed with 2 mL solution of EDC (90 mg) and NHS (55 mg) and incubated at RT for 5 min. 0.036 g Myristate was dissolved in ethanol and added drop wise to the above mixture with continuous stirring for 30 min/RT to obtain a Myristate: CS ratio of 1:9. The mixture was added with ethanol and stirred continuously for another 5 h. The solution was precipitated by increasing the pH to 10 with a 10 M NaOH solution and then centrifuged for 20 min at 5000 g /18 ^o^C. In order to remove the unreacted Myristate, EDC and NHS residuals, the pellet was washed with ethanol followed with distilled water (DW). The precipitated gel was dissolved completely in 2% acetic acid solution and labeled as S1. To find the best storage condition, one mL of S1 nanogel was freeze-dried and stored at RT as S2 sample. Whenever needed, the S2 sample was dissolved in 1 mL of 2% acetic acid with sonication.

*Nanogel Characterization*

*Particle Size Measurement*

The nanoparticles were sputter-coated with a thin layer of gold and scanned by KYKY-EM3200 Scanning Electron Microscopy (SEM) at 25 KV accelerating voltages. The mean particle size and particle size distributions of the nanogels were also determined by Zeta plus Dynamic Light Scattering (DLS) Zeta Sizer Nano-ZS-90 (Malvern Instruments). The mean particle size was measured for 120 replicates and Poly dispersity Index (PdI) were calculated. To disperse the nanoparticles, samples were sonicated in a water bath. The Zeta Plus instrument was used to measure the electrophoretic mobility of the MCS nanogels.

*FT-IR Test*

Spectral analyses of CS, Myristate, MCS, DOX and MCS-DOX carried out by Fourier-transform infrared spectroscopy (FT-IR) using Thermo Nicolet Nexus 870 FT-IR ESP (USA) spectrophotometer. Approximately 100 mg of each of the above dry samples was grounded with KBr and converted to tablets under a pressure of 600 Kg/cm2 by hydraulic press and used for FT-IR analysis.

*Drug Loading and Release*

Method of Wang *et al. *(19) was used to estimate the drug loading and release capacity of the gel. 5.0 mg/mL of MCS nanogel was incubated with 2 mg/mL of DOX. The solution was mixed thoroughly for 24 h/4 °C under shaking condition. The excess free DOX was removed by dialysis using 2 KDa MWCO dialysis bags against 30 mL DW. The DOX loaded MCS was then dialyzed against 30 mL of phosphate-buffered saline, pH 7.4/ 37 °C. At various time intervals 2 mL of phosphate-buffered saline from each dialysis buffer was taken and analyzed by HPLC for the presence of DOX. The buffer taken for analysis was replaced with an equal volume of fresh buffer.

*Determination of Loading Capacity and Release Rate of DOX*

The amounts of the loaded and released DOX were determined by High-performance Liquid Chromatography (HPLC) using a reversed phase column (C18, 5 μM, 250 mm**×** 4.6 mm).

The concentration of DOX was calculated using a standard curve of DOX. Different concentrations of DOX were injected into the column and eluted with sodium lauryl sulfate-Phosphoric acid: acetonitrile of 40:60 ratio with a flow rate of 1 mL/min. The sample solutions were detected with a run time of 7 min for assay. 

The OD of DOX was read at 254 nm. Following equations was used to calculate the loading efficiency (LE) and loading capacity (LC) of the DOX: 

LE = (Total amount of DOX − Free DOX) / Total amount of DOX 

LC = (Total amount of DOX − Free DOX) / amount of MCS

*Synthesis of MCS-GA-CHO Polymer*

Aldehyde-functionalized polymer MCS-GA was synthesized by conjugating

CHO-GA-CHO to MCS-NH2 by the drop wise addition of 0.03 mM MCS nanogel to 15.6 mM GA to obtain the 1:500 ratio. The Schiff base cross-linking involves a reaction between the amine groups of Chitosan and the -CHO groups of glutaraldehyde. The yellow color developed indicates the reaction progress. The excess GA was removed by dialysis of the mixture against 1 L DW/ RT for 24 h using 3 KDa MWCO dialysis bags. The MCS-GA was stored at 4^ o^C after sonication.

*Conjugation of Aminated ssDNA Aptamers to MCS-GA-CHO Nanogels*

To attach the DNA aptamers to MCS-GA-CHO nanogels using glutaraldehyde as a linker, the aptamers were amplified with the NH2–labeled forward and the phosphate-labeled reverse primers. Aminated DNA aptamers were treating with lambda exonuclease in order to get ssDNA. The ssDNA was denatured/ 90 ^o^C for 10 min and then incubated on ice for 15 min to obtain their native folding. 2-4 nM of ssDNA aptamers was added drop wise to 1 mg MCS-GA-CHO nanogels in the total volume of 1mL and incubated for 6 h under shaking condition. In order to monitor the MCS-GA-Apt nanogels, 2 µL of 5 nM Rd was added to the mixture. After 1h shaking, 5 mg Glycine dissolved in 0.5 mL DW was added to the mixture and shacked for another 30 min. 15mg NaBH_4_ dissolved in 1 mL DW was added drop wise to the mixture with continuous shaking for 30 min. The mixture was centrifuged at 12000 g/ 10 min and washed twice with DW and resuspended in BB by sonication and stored/ 4^ o^C. 

*Flow Cytometry Binding Assays*

The affinity of the aptamer conjugated nanogel to the Pcanc cells was evaluated by Flow cytometry. Approximately 10^6 ^live LNCaP and PC-3 cells were washed and resuspend in 100 μL BB. 1 mL MCS-Apt/Rd in BB was incubated with both LNCaP and PC-3 cells for 1 h/ 37 ^o^C in a humid environment with 5% CO_2_. 50 μL from each cell lines were diluted in 1 mL BB and was pipetted into different flow cytometry tubes. The fluorescence intensity of Rhodamin of each tube was determined against the untreated cells alone as a background. 

*DOX-Loading in MCS-GA-Apt Nanogels*

10 mg DOX-HCl powder was dissolved in 1 mL DMSO. 0.5 mL DOX in DMSO was mixed with the MCS-GA-Apt nanogels and incubated for 2 days/4 ^o^C under shaking condition. The mixture was dialyzed against DW using a dialysis tubing with a cutoff of 3 KDa for 24 h to remove the nontrapped DOX.

*In-vitro Cell Viability*

The relative number of the viable cells after treatment was determined by MTT assay. 1×10^4 ^LNCaP cells/well were plated in 96-well plates containing RPMI medium, 10% fetal bovine serum and 1% Pen Strep. After 24 h, the medium was replaced with various concentrations of DOX in RPMI medium containing 1% Pen Strepwithout FBS. After 24 h, 20 μL of MTT (3 mg/mL) was added to each well and the plates were incubated/ 37 °C for 5 h. The cells were then centrifuged at 700 g for 15 min and the supernatant was discarded. The cell pellets were dis­solved in 100 μL of DMSO and the OD_570_ was measured. The untreated cells were considered as a control. The percentage of viability was determined by the following formula:

%Cytotoxicity = 1- (mean absorbance of toxicant / mean absorbance of negative control) **×** 100 

%Viability = 1- % Cytotoxicity

The MTT assay was repeated with 0-200 µM of each of MCS, DOX-loaded MCS (non-targeted) and MCS-GA-Apt (targeted) nanogels and the percentage of viability was determined as above. 

*Gel Electrophoresis*

To confirm aptamer conjugation, MCS-GA-Apt nanogels were loaded into the 1% agarose gel along with the molecular weight ladder. The electrophoresis was performed at 50V and the gel was stained with 0.5 mg/mL Et. Bromide. 

*Statistical Analysis*

The statistical differences between the test and control samples were determined by Analysis of Variance: ANOVA One-Way with P < 0.05. Values are considered as means ± standard deviation for at least three independent experiments by Tukey analysis.

## Results

*SELEX Procedure*

**Figure 1:**
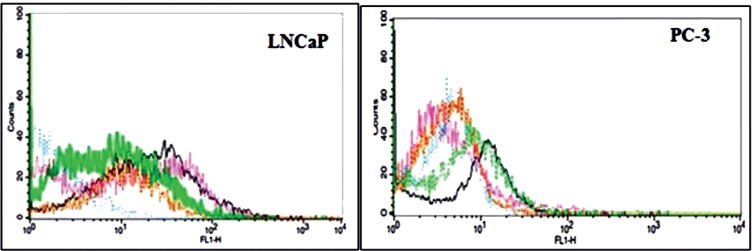
Comparison of flourescent intensity of the finally selected aptamers with the original library binding to LNCaP and Pc3 cell lines. The blue line is for Library, the purple line is for 4^th^ SELEX , the green line is for 7^th^ SELEX, the black line is for 9^th^ SELEX and the brown line is for 10^th^ SELEX

The fluorescence intensity of the LNCaP cells was increased to 49% in the 4^th^ SELEX and 79% in the 10^th^ SELEX compared to the control PC-3 cell lines (Figure 1). Since there was no significant difference in the fluorescence intensity of SELEXes 9^th^ and 10^th^, the SELEX procedure didn’t continue beyond the 10^th^ round of SELEX. The binding affinity of the selected aptamer pool was above 79% compared to the12.5 % of the initial library. 

*Sequencing of Enriched Pools*

**Figure 2:**
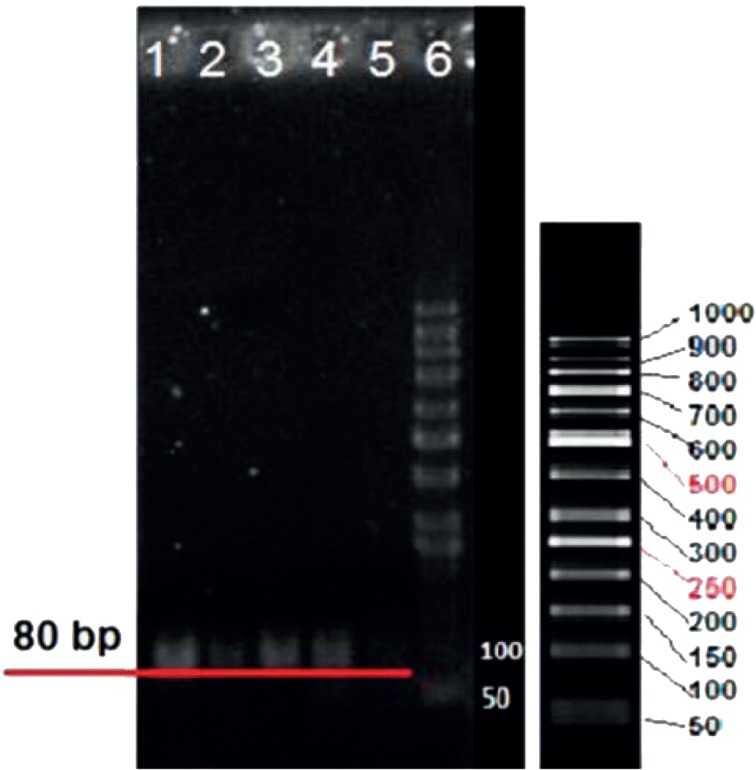
Agarose gel electrophoresis showing colony PCR product of selected aptamers with high affinity to LNCaP cells. Lanes 1-5 belong to 80 bp cloned selected aptamers. Lane 6 belongs to 50 bp ladder

Colony PCR product of 80bp selected aptamers with high affinity to LNCaP cells on agarose gel electrophoresis is exhibited in Figure 2. The nucleotide sequences of the highest affinity binding DNA aptamer to LNCaP cells is given below. The underlined nucleotides belong to the variable region of the aptamer flanked with the known sequences at the either sides.

5`CATCCATGGGAATTCGTCGACCCTGCAGGCATGCAAGCTTTCCCTATAGTGAGTC

GTATTACGAGCTCGAGCCTAGGCAG3`

The dissociation constant (K_d_) for the highest binding aptamer was 34 ± 3.8 pM. This aptamer was conjugated to nanogel.

*CS Nanogels and Particle Size*

**Figure 3:**
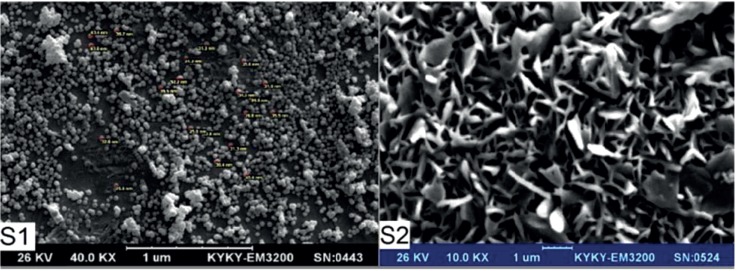
SEM of MCS nanogels for Samples S1 and S2.

**Figure 4:**
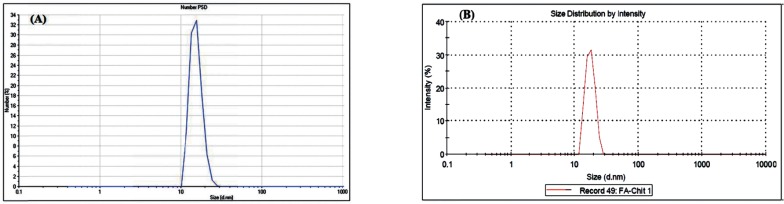
DLS Results for S1 MCS nanogel: (A) Zeta Potential Distribution. (B) Size Distribution Report by Intensity

**Figure 5:**
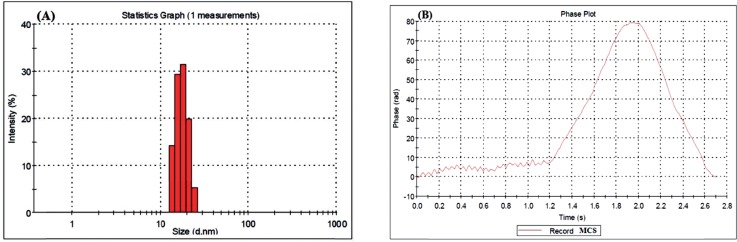
DLS Results for S1 MCS nanogel: (A) Statistics Graph. (B) Phase plot curve

Table 1. shows the results of the nanogels characterized by SEM and DLS. Figures 3-5 show the particle size of MCS nanogels characterized by SEM and the results of the DLS analysis. As shown in Figure 3. the S2 nanogel appeared as an unexpected rod shape and hence we didn’t continue for further analysis. The S1 nanogel had a spherical shape with dense surface. The average diameter of the particles were of about 15-24 nm. The Zeta potential of the nanoparticles determined for S1 samples in DW/ 25 °C is given in Table 1 and Figure 4 and 5. The size distribution for the S1 MCS nanogels was found to be 15–25 nm (Figure 4B), which is also confirmed by the SEM images. The polydispersity (PdI) obtained for S1 was 0.016 (Table 1).

*FTIR Analysis*

**Figure 6:**
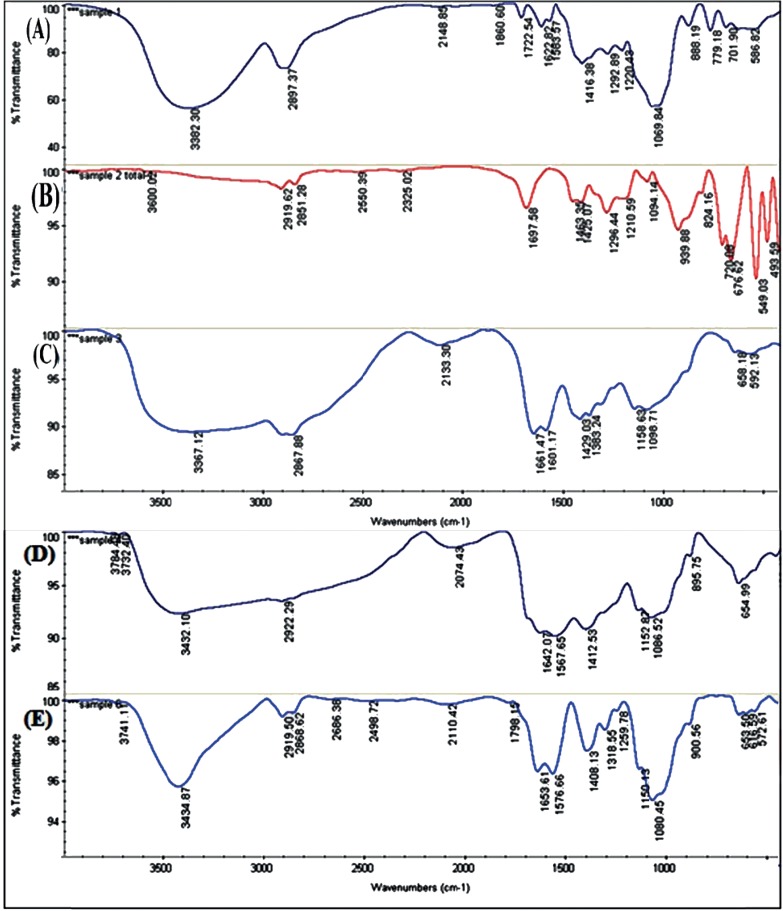
FTIR spectra of (A) DOX, (B) Myristate, (C) Non-modified CS, (D) MCS and (E) DOX loaded MCS

The structure of CS, DOX, MCS, Myristate and DOX-MCS characterized by FT-IR spectrometry is shown in Table 2 and Figure 6 (A-E). 

*In-vitro Drug Loading and Release*

**Figure 7:**
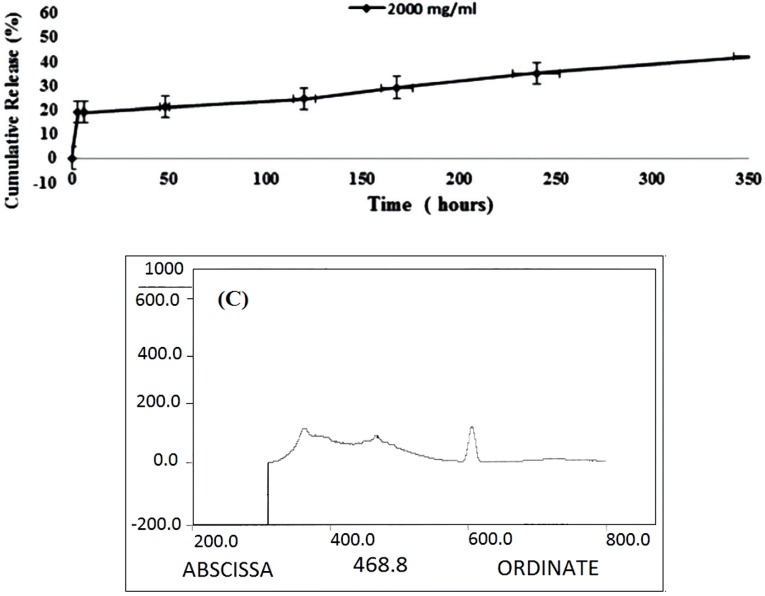
Release pattern of 2 mg/mL DOX loaded 5 mg/mL MCS during 360 h (15 days)/ 37 ^o^C

The results of *in-vitro* drug release of DOX-MCS are shown in Figure 7 and Table 3. Approximately 18.5% DOX was released within the first 3 h and reached to 40% after 

360 h/ 37 ^o^C. 

*Characterization of MCS-GA-Apt/Rod /Gly polymer*

*Fluorimetric Assay*

**Figure 8:**
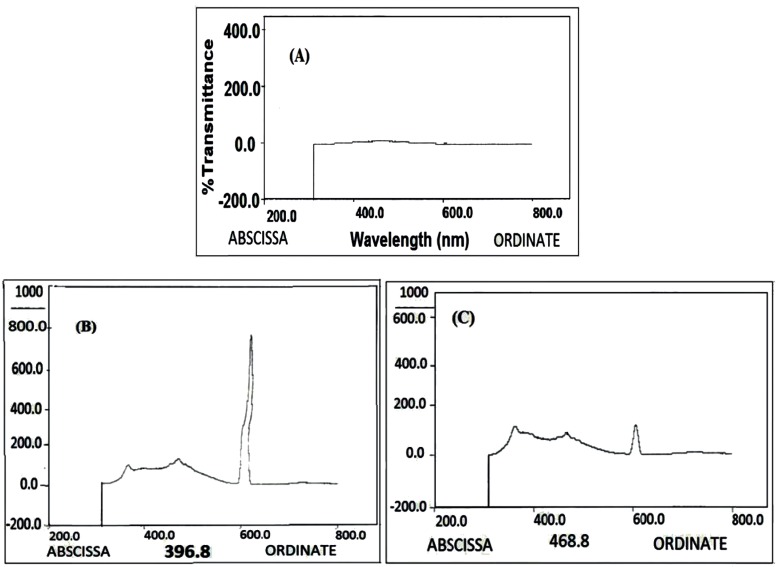
The fluorimetric assay for binding affinity of Rd and Glycine to MCS-GA-CHO, (A) MCS-GA-CHO without Rd (B) MCS-GA-CHO with Rd before washing (C) MCS-GA-CHO with Rd after washing

The interaction of Rod with MSC-GA-CHO/ Gly to form MSC-GA- Rod/ Gly polymer was detected by fluorimetric assay (Figure 8). Figure 8A shows the nanogel alone, where there is no pick at 534 nm. When Rod and Gly were added to the gel, a sharp peak appeared at 534 nm (Figure 8B). The peak was retained even after washing the gel (Figure 8C).

*Flow Cytometric Analysis*

**Figure 9:**
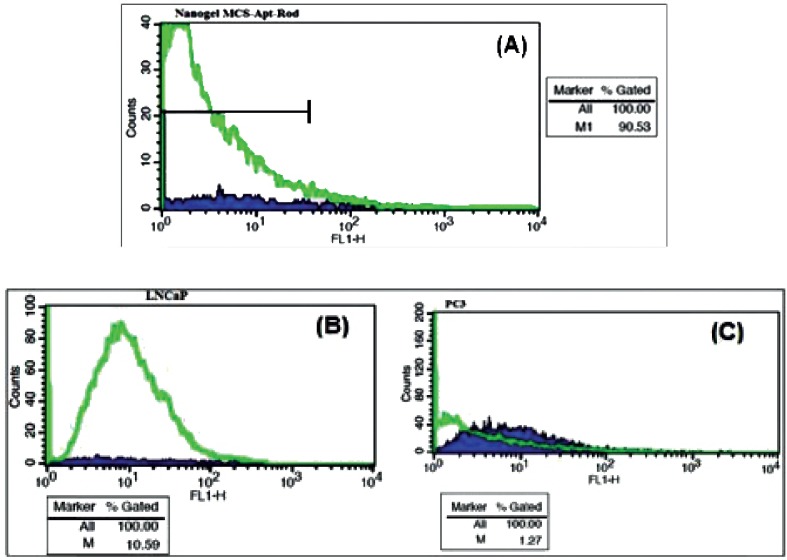
Flow-cytometric assays of conjugated NH2-labeled ssDNA aptamer to (A) MCS-GA-Rd nanogels alone, (B) MCS-GA-Rd nanogels binding to the LNCaP cell lines and (C) MCS-GA-Rd nanogels binding to the PC3 cell lines

The results of Flow cytometry are illustrated in Figure 9A-C*.* The fluorescence intensity of Rd binding to MCS-GA-Apt was increased up to 90% with increasing aptamer concentration up to 4 nmol (Figure 9A). The binding of the NH2-labeled ssDNA aptamer conjugated with MCS-GA to the LNCaP and PC-3 cell lines showed 10 fold higher affinity to the LNCaP cells compare to that of PC-3 cells (Figure 9B and C).

*Agarose Gel Electrophoresis*

**Figure 10:**
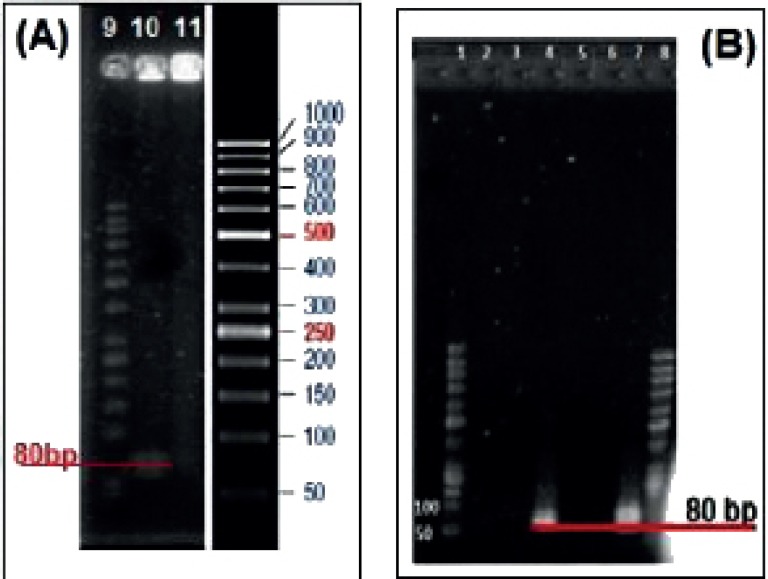
Agarose gel electrophoresis of the conjugated aptamer to MCS-GA nanogels. Lanes 1, 8 and 9 are ladder. Lanes 2 and 3 are unlabeled Apt with MCS after washing. Lane 4 is unlabeled Apt only. Lanes 5 and 6 are FITC labeled Apt with MCS after washing. Lane 7 is FITC labeled Apt only. Lane 10 is unwashed NH2 labeled Apt with MCS and Lane 11 is washed NH2 labeled Apt

The results of conjugation of aptamer to nanogel are exhibited on agarose gel electrophoresis (Figure 10). Wells 4 and 7 were loaded with unlabeled and FITC labeled aptamers respectively. Lanes 2 and 3 belong to MCS treated with unlabeled aptamer whereas lanes 5 and 6 were for MCS treated with FITC labeled aptamer (Figure 10A). Wells 10 and 11 were loaded with MCS treated with 5› NH2-aptamer (Figure 10B). All samples were washed preloading except the sample loaded with well 10 which was retained unwashed.

*In-vitro Cytotoxicity*

**Figure 11:**
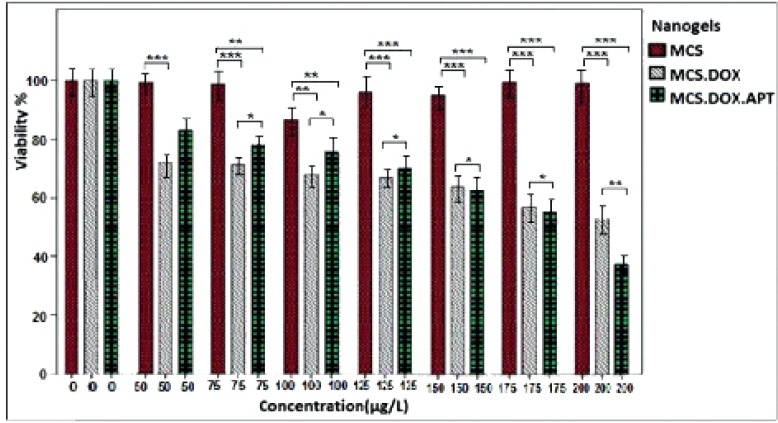
MTT assay for estimation of cytotoxic effect of different concentrations of free MCS, MCS-DOX and Apt-MCS-DOX on viability of LNCaP cell line. * indicates a p<0.1, ** indicate a p<0.01, *** indicate a p<0.001, compared with nanogel

**Figure 12:**
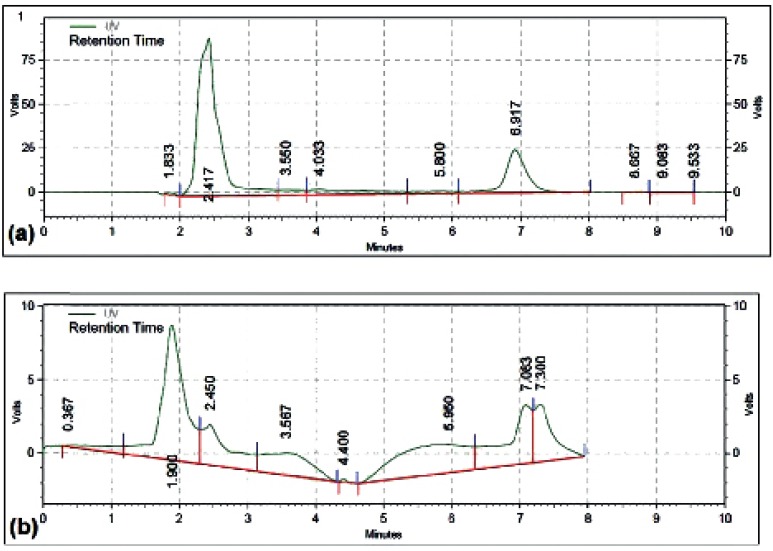
HPLC analysis of DOX release. (a) HPLC chromatograms of the DOX released from Apt-MCS-DOX, (b) HPLC chromatograms of free DOX. DOX released from nanogel revealed one pick with a retention time of 7 min. whereas free DOX exhibited 2 picks with retention times of 7.08 and 7.30

The cytotoxic effect of different concentrations of free DOX, MCS alone, MCS-DOX and Apt-MCS-DOX on the viability of LNCaP cell are shown in Figure 11. The DOX loaded to nanogel showed more stability (Figure 12a) than the free DOX (Figure 12b). 

## Discussion

Many investigators are working on targeting of different delivery systems to the cancer cells. In most of these investigations antibodies have been used as a targeting agent. In the present study, due to several advantages of aptamers over antibodies, a nanoparticles, MCS, was prepared and conjugated with a specific aptamer developed to LNCaP cells and loaded with DOX as an anti-prostate cancer drug. The complex was applied to LNCaP and PC-3 cell lines to study for it’s binding affinity. To obtain specific aptamer, Cell-SELEX was carried out using LNCaP cell lines as a target and PC-3 cell line for counter SELEX. 

**Table 1.** SEM and DLS of MCS nanogels. S1) Myristate: CS at a ratio of 1:9

MCS	**S1**
Mean Particle size by SEM	15.64
DLS Record Number	120
Zeta potential (mV)	20.7± 2.02
Z-Average size (d. nm)	24.48 ± 5.09
PdI	0.016

**Table 2.** Main peak wave numbers of FTIR spectra obtained from non-modified CS, DOX, MCS and DOX loaded MCS

**Chitosan(CS)**	**Cm** ^-1^	**MCS**	**Cm** ^-1^
O–C–O scissors	595/658	The stretching vibration of the O-H group is overlapped to the N-H stretching band	3432
The C–O stretching absorption peak of the secondary hydroxyl group	1098	C–H stretching vibrations	2922
C–O–C in glycoside linkage	1158	C–C stretching vibrations P-O-R	1412
N-H bending	1601	P–O–R esters	895
C=O carbonyl stretching vibrations of the esteemed group	1661	O–C–O scissors	654
**DOX**	**Cm** ^-1^	**MCS-DOX**	**Cm** ^-1^
O-H stretching	3382	The stretching vibration of the O-H group is overlapped to the N-H stretching band	3435
C–H stretching vibrations	2897	C–H stretching vibrations	2919
C–C stretching vibrations	1416	C=O carbonyl stretching vibrations of the esteemed group N-H bending C–C stretching vibrations	1653 1575 1408
NH2 & N–H wagging	779	P-O-R	1080
N–H wagging	701	O–C–O scissors	653

**Table 3.** DOX release of MCS nanogels upon dialysis

DOX (mg/ml)	**MCS:DOX**	**Loading Capacity** **(DOX/mg MCS)**	**Dox Loaded** **(mg/L)**	**Loading Efficiency** ** (%)**
2.0	0.4 : 1	39.63	1981.46	99.08
5.0	1 : 1	96.5	4825	96.5

Flow cytometry analysis revealed that after 10 rounds of SELEX, the DNA aptamers showed 79.83% improvement on binding affinity to the LNCaP cells compared to the original library. The enriched DNA aptamers was cloned into pTG19-T plasmid and positive clones for DNA aptamers were sequenced. Positive clones were amplified and tested for binding affinity individually. The clone named Apt 1 exhibited the highest affinity and this clone was used for conjugation to MCS nanogels.

CS as a non-toxic, biodegradable and biocompatible polymer has developed for a new drug delivery system(Thakare et al. 2015). The Myristic treatment of CS nanoparticles can improve their stability and applicability in packing drug and controlled drug release. The Myristate: CS was prepared in a ratio of 1:9 in order to provide extra amino groups for reacting with other groups such as carboxyl, aldehyde and etc. The other ratios such as 1:6 and 1:4 was also examined. In these ratios the glutaraldehyde was not sufficiently attached to the nanogel. The structures of CS, DOX, MCS, Myristate and DOX-MCS are characterized by FT-IR spectrometry. The DOX peak at 1416 cm^−1^ was seen in DOX loaded MCS nanogels at 1408 cm^−1^ confirming the presence of DOX in the MCS-DOX nanogels (Figure 6A and E). The results reported by R. Jayakumar (20) and Kayal (21) are in support of our findings. A shift from 779 to 900 cm^−1^ related to N–H wagging, is due to the presence of intermolecular hydrogen bonding between the DOX and MCS loaded with DOX. Following the chemical modification of CS to MCS, the absorption band of N-H bending at 1601 cm^−1 ^of CS is moved to a lower position of 1567 cm^−1^ in MCS and shifted to 1576 cm^−1^ in MCS-DOX (Figure 6C, D and E). This could be attributed to cross linking between Myristate and CS producing amid groups. The characteristic peaks from the N–H and O–H stretching, vibration bands are overlapping on a wide strong peak at 3367 cm^−1^ in CS and a sharp and high energy at 3432 cm^−1^ in MCS and 3434 cm^−1^ in MCS-DOX structure (Figure 6C, D and E). This could be due to the hydrogen bonding between –OH and –NH groups. Mitra *et al.,* have reported similar results (22). Sonication didn’t affect the loading of DOX on MCS.

The drug loading capacity was increased with increasing DOX concentration, whereas the loading efficiency didn’t increase beyond 1:1 ratio of MCS: DOX. We obtained similar results up on loading Phosphorylated Chitosan (PCS) with DOX. The 24h incubation time enhanced the loading amount of DOX to about 99%. (Table 3). Our findings were better than those obtained by Jayakumar (20) where the loading efficiency was reported to about 26% in Chitin-DOX after 5h incubation. The loading capacity of SN38, an anticancer drug on CS-SN38–Apt nanoparticles was reported to be about 7.1% (23). The release profile of DOX from DOX loaded nanogels/ 37 °C was slow and time dependent. Yang *et al.,* have reported 11% of DOX release from graphene oxide within the first 5 h (24). The release percentages of DOX reported by Xu *et al.,* of the four samples were about 73.1%, 60.5%, 47.7% and 36.8%, after 10 h (25). Their data indicated that the behavior of DOX release was related to the DOX concentration during the drug encapsulation.

Since the finding of this research will finally be applied for the *in-vivo* delivery of the drug to the Pcanc cells in the animal model, therefore the slow release of DOX will provide us the continuous release of DOX to the target. This can further increase the advantage of our nanogel as a better therapeutic agent. In a study reported by Wu, *et al.,* DOX was loaded to PEG/PEI-Fe_3_O_4_ (PEGylated polyethyleneimine - Iron oxide) up to 85% and nearly 81% DOX was released from DPNTS (drug-loaded, pH sensitive, nano-magnetic targeted system) within 72 h/ pH 4.5, but the *in-vivo* rate of release was only 28 % (26).

Glutaraldehyde was used as a linker between MCS and Rod/Apt which simply binds to the MCS nanogels and Rod/Apt or Gly by direct interaction between –CHO from glutaraldehyde and -NH2 groups from Rod/Apt. The Schiff base cross-linking, which involves a reaction between the -CHO groups and the amine group was fixed by the addition of NaBH_4_ solution to the nanogel mix. As demonstrated in Figure 8C, when the gel was treated with Rod and then washed, the peak was retained even after washing which indicates the successful binding of Rod to the nanogel. Gly was used to block the remaining free -CHO groups on glutaraldehyde and also help the nanogel to be dissolved at physiological pH. The presence of bands on lane 4 and 7 indicate the migration of unlabeled and FITC labeled aptamers through the gel respectively (Figure 10). Both the aptamers served as a positive control. When these aptamers were treated with MCS and then washed, no band appeared either in the wells or within the gel (lanes 2, 3, 5 and 6). Since the mixtures were washed before loading into the wells, it indicates that the aptamers didn’t link to MCS nanogel and were eliminated upon washing. Wells 10 and 11 were loaded with MCS conjugated with 5› NH2-aptamer before and after washing respectively. A band of aptamer was appeared in lane 10 upon staining with Et. Bromide but was absent in lane 11. This means that the extra unattached aptamers migrated to the gel. After washing, there was no extra aptamer to move through the gel on well 11 to show any band. Since MCS conjugated with aptamers could not move into the gel due to their large size, this is an evidence for Apt-MCS conjugation. Similar agarose gel electrophoresis experiments were carried out with Sayari *et al.,* to prove the conjugation of MUC1 DNA aptamer to CS (23). Guo *et al* applied Urea PAGE staining with Et. Bromide to confirm the conjugation of aptamer in the presence of EDC to NP surface.  NP alone did not show any band on PAGE gel whereas a band observed at the loading site with conjugated aptamer on the NP surface (10). Cheng *et al.,* also confirmed aptamer conjugation using 10% TBE (Tris/ Borate/ EDTA)-Urea PAGE (18).

The cytotoxic effect of different concentrations of free DOX, MCS alone, MCS-DOX and Apt-MCS-DOX on LNCaP cell viability was estimated using MTT assay. The IC_50_ of free DOX was estimated to be about 0.15 mM and the concentration of the nanogels used in the experiment was from 50 µM to 200 µM. As shown in Figure 11. incubating the cells with MCS alone, didn’t show any cytotoxicity whereas application of MCS-DOX and Apt-MCS-DOX showed a significant (p ˂ 0.001) lethal effect on the LNCaP cell lines. There are several reports correlated to reduced toxicity of conjugates such as PEG derivated conjugates like EZN-2208, or hMN-14 conjugates with SN38 drug compared to the free drug (27). Compare to free DOX, Apt-MCS-DOX revealed longer storage stability at 37 ^o^C which enhances the efficacy of the binding of nanogel to the cell surface of Pcanc (Figure 12). This would allow accumulation of sufficient drug with a developed lifetime of Apt-MCS-DOX on surface of targeted cancer cells. Several studies previously reported similar uptake of DOX using an aptamer mediated delivery (12). Cao *et al.*, showed the binding of HRP-CS nano particle to the targeted cell surface (28). Sayari *et al*. reported enhanced and selective uptake of CS-SN8 conjugated with aptamer in comparison with non-decorated NPs on HT-29 cell line while there was no significant difference between them in cellular uptake with CHO cell lines (23).

## Conclusion

Loading DOX in Apt-MCS conjugate increased drug efficacy via diminishing toxicity and targeted delivery to the LNCaP cells. Our results can lead to a simple and applicable technique for an appropriate drug carrier in the treatment of cancerous cells. Substitution of aptamer for monoclonal antibodies for early diagnosis of and targeted drug delivery to the tumor cells is a further advantage of this research.
